# Long distance dispersal and vertical gene flow in the Caribbean brooding coral *Porites astreoides*

**DOI:** 10.1038/srep21619

**Published:** 2016-02-22

**Authors:** Xaymara M. Serrano, Iliana B. Baums, Tyler B. Smith, Ross J. Jones, Tonya L. Shearer, Andrew C. Baker

**Affiliations:** 1 Division of Marine Biology and Ecology, Rosenstiel School of Marine and Atmospheric Science, University of Miami, 4600 Rickenbacker Causeway, Miami, FL 33149, USA; 2 Department of Biology, The Pennsylvania State University, 208 Mueller Laboratory, University Park, PA, 16802, USA; 3 Center for Marine and Environmental Studies, University of the Virgin Islands, #2 John Brewer’s Bay, St. Thomas, USVI 00802-9990, USA; 4 Australian Institute of Marine Science, The UWA Oceans Institute, 35 Stirling Highway, Crawley, WA 6009, Australia; 5 Georgia Institute of Technology, School of Biology, 310 Ferst Dr., Atlanta, GA 30332, USA

## Abstract

To date, most assessments of coral connectivity have emphasized long-distance horizontal dispersal of propagules from one shallow reef to another. The extent of vertical connectivity, however, remains largely understudied. Here, we used newly-developed and existing DNA microsatellite loci for the brooding coral *Porites astreoides* to assess patterns of horizontal and vertical connectivity in 590 colonies collected from three depth zones (≤10 m, 15–20 m and ≥25 m) at sites in Florida, Bermuda and the U.S. Virgin Islands (USVI). We also tested whether maternal transmission of algal symbionts (*Symbiodinium* spp.) might limit effective vertical connectivity. Overall, shallow *P. astreoides* exhibited high gene flow between Florida and USVI, but limited gene flow between these locations and Bermuda. In contrast, there was significant genetic differentiation by depth in Florida (Upper Keys, Lower Keys and Dry Tortugas), but not in Bermuda or USVI, despite strong patterns of depth zonation in algal symbionts at two of these locations. Together, these findings suggest that *P. astreoides* is effective at dispersing both horizontally and vertically despite its brooding reproductive mode and maternal transmission of algal symbionts. In addition, these findings might help explain the ecological success reported for *P. astreoides* in the Caribbean in recent decades.

Variation in life-history characteristics, such as reproductive mode and larval type, has often been used to predict patterns of larval dispersal and connectivity in marine invertebrates^1^. Scleractinian corals are excellent study models, as they exhibit a variety of life-history and reproductive strategies that can directly influence their potential for dispersal^2^. Brooded larvae are more advanced in their development when released than larvae from broadcast spawners, and are therefore competent to settle within hours^3^,^4^. In addition, algal symbionts (*Symbiodinium* spp.) are transmitted directly to brooded offspring^3^ (i.e., maternal transmission), which may constrain the colonization and post-settlement survival of the coral offspring. For example, brooding corals may be limited in their ability to settle outside the direct parental range if depth-specific symbionts are transferred. In contrast, larvae from broadcast spawners – in which gametes are fertilized in the water column – usually require 5–7 days of development to achieve competency (reviewed in Harrison and Wallace^5^). In addition, algal symbionts are not generally present in the eggs of broadcast spawning coral species and must be acquired from the environment, often several days after settlement (e.g., Coffroth *et al.*^6^). Together, these characteristics are thought to facilitate greater dispersal in broadcast spawners compared to brooding species. However, no studies have yet examined the potential for horizontal vs. vertical (i.e., across depths) dispersal for a Caribbean brooding coral.

*Porites astreoides* (Lamarck, 1816) is a common coral species found throughout the Caribbean, occurring over a wide range of depths and habitats^7^,^8^ down to 70 m^9^. It occurs as two color morphs, with the yellow/green morph generally observed in shallower waters than the brown morph, although both are often found side by side^10^,^11^. In addition, *P. astreoides* undergoes internal fertilization, releasing semi-mature planulae monthly from January to September^4^,^12^ and has an unusual mixed breeding system, in which half of the colonies are hermaphroditic and the other half are female^13^. Algal symbionts are present in brooded *P. astreoides* larvae when released^13^ and appear to follow strong zonation patterns along depth gradients of 2–25 m in Panama^14^, the Bahamas^14^, Belize^15^ and Curacao^16^. Finally, *P. astreoides* appears to be the only scleractinian coral species that is becoming a more prominent component of coral reef communities throughout the Caribbean^17^. A comparison of historical data collected from 1974–1992 with photoquadrats from 2003–2004 revealed that the relative percentage of cover of *P. astreoides* has increased from <20% to 50% in shallow habitats spanning over a 4,000 km arc of the Caribbean^17^.

In a previous study (Serrano *et al.*^18^), we undertook a comprehensive analysis of population genetic structure in the broadcast spawning coral species *M. cavernosa*, in both horizontal and vertical directions. Significant genetic differentiation with depth was observed in Florida (Upper and Lower Keys), but not in Bermuda or the U.S. Virgin Islands (USVI), despite high levels of horizontal connectivity between all three of these geographic locations at shallow depths. These observations strongly suggest that horizontal connectivity is much greater than vertical connectivity for *M. cavernosa*. However, whether these patterns are consistent across other scleractinian corals from the Caribbean is not yet understood.

The current study represents the first assessment of genetic connectivity for the coral species *Porites astreoides* in the tropical and subtropical western Atlantic. We aimed to examine the extent to which the life-history and reproductive strategies of *P. astreoides* may influence its patterns of larval dispersal and gene flow in both horizontal and vertical directions, and help explain this species’ ecological success in the Caribbean^17^. To accomplish this, we collected samples of *P. astreoides* from sites in the Upper Keys, Lower Keys and Dry Tortugas (within Florida), Bermuda and the USVI, at three depth zones [denoted as “shallow” (≤10 m), “mid” (15–20 m and “deep” (≥25 m), see Supplementary Table S1 and Fig. S1]. We then used a combination of newly-developed and existing DNA microsatellite loci for *P. astreoides* to evaluate patterns of connectivity among geographic locations (long-distance horizontal dispersal), among reefs within a geographic location (within Florida), and among depths in each region (vertical dispersal). We also tested whether connectivity patterns could be correlated with differences in color morph (yellow/green vs. brown). Finally, we used a combination of denaturing gradient gel electrophoresis and quantitative PCR in a subset of corals to assess patterns of depth zonation in algal symbionts (*Symbiodinium* spp.) if any, and test whether maternal transmission of algal symbionts might limit effective vertical connectivity for *P. astreoides.*

## Results

### Multi-locus genotyping and tests of Hardy Weinberg Equilibrium

Our genetic analysis of 660 *P. astreoides* samples yielded 590 unique multi-locus genotypes (Supplementary Table S1), suggesting ~10% clonality, either as a result of asexual reproduction or insufficient resolution in our markers to distinguish these individuals. Most clones, however, were confined to a single sampling location (within <1 km) except in two cases, both at two different mid-depth sites within the Upper or Lower Keys. Tests of Hardy Weinberg Equilibrium (HWE) for each of the 15 combinations of region/depth individually revealed that all 8 loci are largely in HWE, with only 5.8% of 120 tests showing significant deviations from HWE after FDR correction (Supplementary Table S2). Individual inbreeding values were generally low (F_i_ mean = 0.01; 95% confidence interval = 0–0.04), as well as null allele frequencies (which ranged between 0.03–0.06 across loci and populations, see Supplementary Table S3).

### Assessment of vertical vs. horizontal connectivity

**Figure 1:**
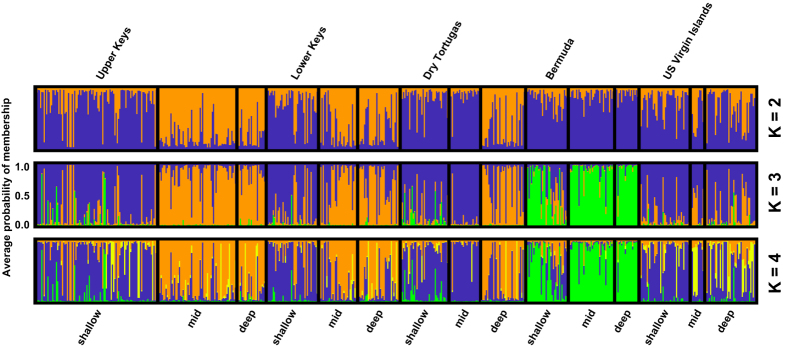
*Porites astreoides* population structure across regions [Upper Keys, Lower Keys and Dry Tortugas (within Florida), Bermuda and the U.S. Virgin Islands] and depths [shallow (≤10 m), mid (15–20 m) and deep (≥25 m)]. Bar graphs show the average probability of membership (y-axis) of individuals (N = 590, x-axis) in K = 2 to K = 4 clusters (shown in ascending order) as identified by STRUCTURE. Samples were arranged in order of increasing depth within region.

**Figure 2:**
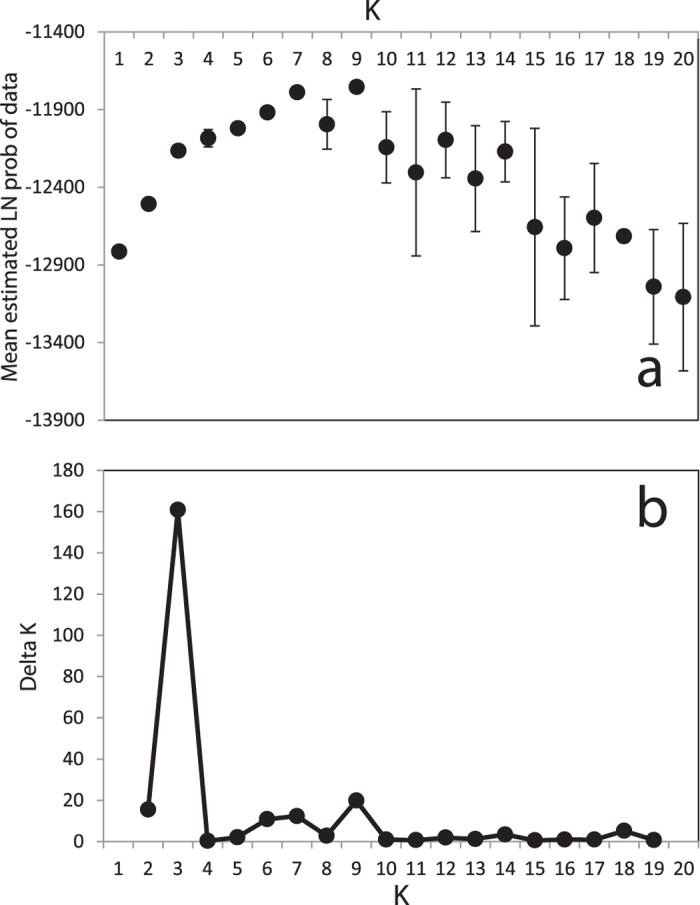
Mean log-likelihood of K (**a**) and Delta K (**b**) values for STRUCTURE analysis of *Porites astreoides* samples. Values of K = 1–20 were tested by running 3 replicate simulations for each K (error bars in upper figure indicate variance among replicates).

Patterns of genetic subdivision for *P. astreoides* showed strong support for three clusters (Figs 1 and 2a,b and Supplementary Table S5 and Fig. S2) that correlate with depth in Florida (shallow vs. deep), and with geographic distance (Bermuda vs. Florida or the USVI). Within Florida, significant differentiation with depth was observed in all three regions. The largest differentiation occurred in the Upper Keys, where most of the individual colonies at intermediate and deep depths (≥15 m) were assigned with high probabilities of membership to the deep cluster (depicted in orange, Fig. 1). Conversely, the Dry Tortugas exhibited significant differentiation with depth, but at depths ≥25 m, and with only about half of the colonies at this depth assigned with high probabilities of membership to the deep cluster. Finally, STRUCTURE showed difficulty in assigning some Floridian individuals to either cluster, indicating some degree of admixture, perhaps as a result of interbreeding between shallow and deep colonies. In addition, a subset of individuals from both shallow and deep habitats displayed high probabilities of membership to the opposite depth to the one they were collected from, especially in the Lower Keys. These findings suggest that these individuals might be immigrants to their assumed populations or have recent immigrant ancestors. Finally, in contrast to significant depth differentiation in Florida, high levels of gene flow were observed among depths in the USVI and Bermuda. However, whereas corals from all depths in the USVI shared the common shallow cluster present in Florida, the “local” Bermudian cluster (depicted in green, Fig. 1) dominated across all depths within this geographic location.

**Figure 3:**
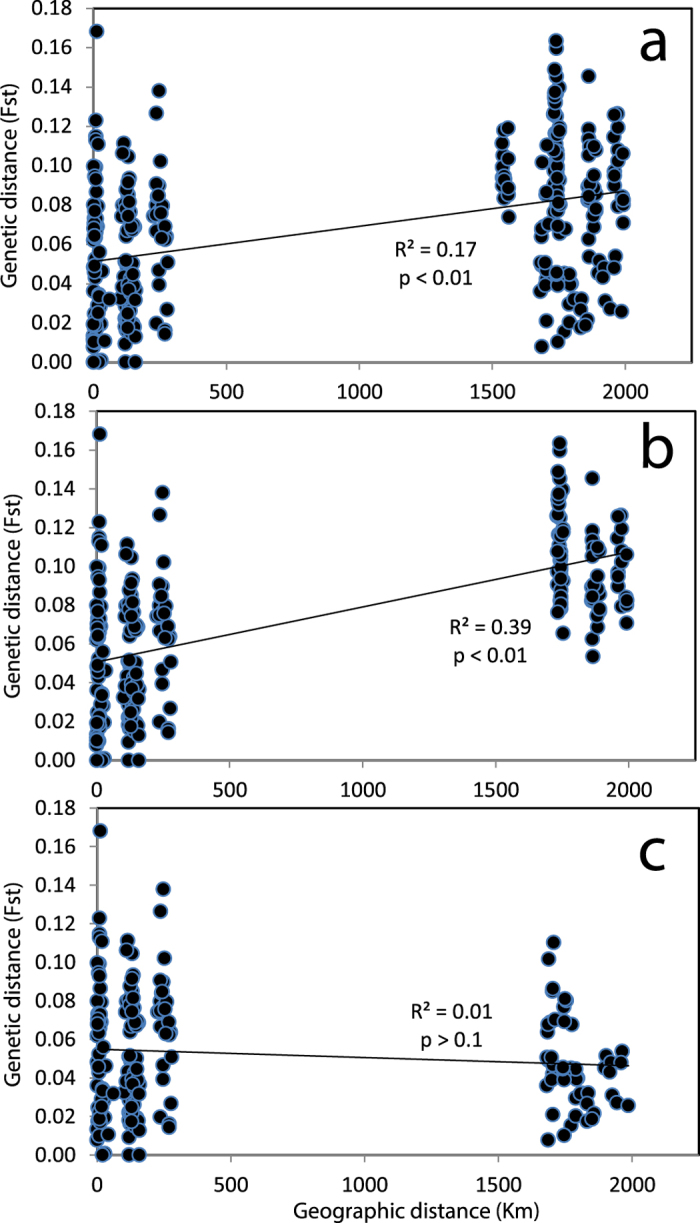
Isolation-by-distance patterns in *Porites astreoides*. Geographic distance explained 17% of the variation in genetic distance (F_ST_) across all sampling sites (R^2^ = 0.17, P < 0.01, Fig. a), 39% of the variation in genetic distance when the U.S. Virgin Islands sites were excluded (R^2^ = 0.39, P < 0.01, Fig. b), and none of the variation when Bermuda sites were excluded (R^2^ = 0.01, P > 0.05, Fig. c).

**Figure 4:**
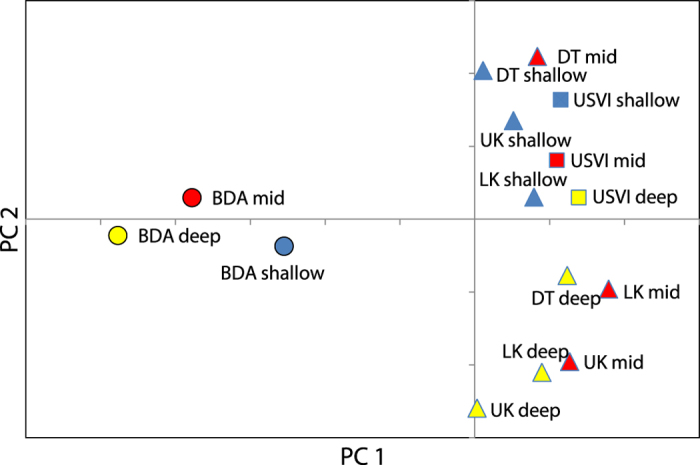
Principal Component Analysis (PCA) of allele frequency covariance in *Porites astreoides* populations. 14 of 79 axes were retained, explaining 100% of the cumulative variance. Plotted are the first and second axes explaining 38.59% (P < 0.01) and 21.28% (P < 0.05) of the variance, respectively. Axes cross at 0. The different shapes denote each of the 3 geographic locations sampled in this study (Florida, Bermuda and U.S. Virgin Islands), whereas different colors denote each of the 3 depths under comparison [shallow (≤10 m), mid (15–20 m) and deep (≥25 m)]. UK = Upper Keys, LK = Lower Keys, DT = Dry Tortugas, Bermuda = Bermuda and USVI = U.S. Virgin Islands.

**Figure 5:**
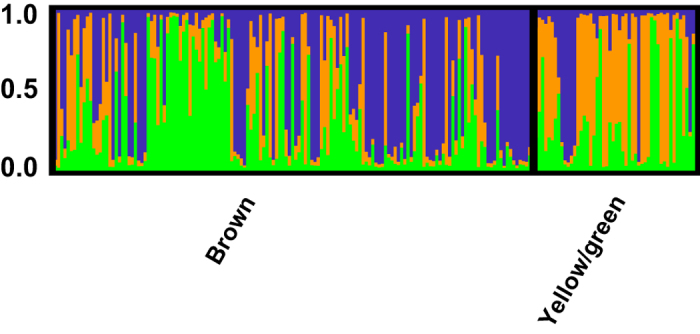
*Porites astreoides* population structure by color morph (yellow/green or brown). Bar graphs show the average probability of membership (y-axis) of individuals (N = 200, x-axis) in K = 3 clusters as identified by STRUCTURE.

Overall, no genetic structure was observed among shallow sites in Florida and the USVI (Fig. 1), suggesting a high degree of horizontal connectivity among sites separated by >1,700 km within the Caribbean and northwest Atlantic. Bermuda, however, appears relatively isolated, with only a few individuals at the shallow inshore site clustering with shallow corals from Florida and the USVI (Fig. 1 and Supplementary Fig. S3). Isolation-by-distance analyses confirmed these results (Fig. 3a–c), with 39% of the variation in genetic distance explained by geographic distance between Florida and Bermuda, compared to 0% explained by geographic distance between Florida and the USVI. Pairwise F_ST_ estimates were also in agreement (Table 1), as F_ST_ values were largest for Bermudian populations compared to any of the populations from Florida or the USVI regardless of depth. Finally, Principal Components Analysis (Fig. 4) also suggested that populations from Bermuda were genetically isolated from Florida or the USVI (PC 1, explaining 39% of the variance) and that habitats clustered together by depth within Florida (PC 2, explaining 21% of the variance). All populations within the USVI (regardless of depth) clustered together with the common shallow population present in Florida. Finally, since *P. astreoides* is known to occur as two color morphs (yellow/green and brown), this information was recorded whenever possible (N = 200). Our findings, however, show that genetic subdivision was not associated with color morph type (Fig. 5), suggesting that both color morphs constitute a single species (see Gleason^10^).

### Algal symbiont characterization

Scleractinian corals depend critically on the mutualistic association with dinoflagellate endosymbiotic algae in the genus *Symbiodinium*, consisting of at least 9 phylogenetically distinct clades (A-I)^19^. To date, most of the depth-generalist Caribbean coral species studied have been found to exhibit marked patterns of depth zonation in *Symbiodinium*^9^. However, intraspecific variation tends to be greatest in shallower reefs (1–8 m), where some species have been shown to host up to five distinctive symbionts (e.g., *Orbicella faveolata*^15^). Furthermore, in some cases, the limits of reef coral vertical distribution have been correlated with the photophysiological capacity of the symbionts hosted (e.g., Iglesias-Prieto *et al.*^20^).

**Figure 6:**
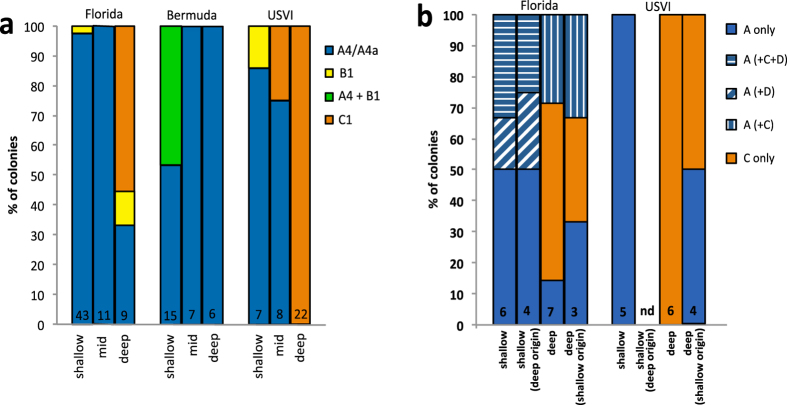
*Symbiodinium* types detected in a subset of *Porites astreoides* corals from shallow (≤10 m), intermediate (15–20 m) or deep (≥25 m) depths, using denaturing gradient gel electrophoresis (**a**) versus high-sensitivity quantitative PCR (**b**). In (**b**), potential immigrants or individuals with immigrant ancestors were identified in STRUCTURE as having a probability of membership >0.90 to the deep cluster [denoted as ‘shallow (deep origin)’], or as having a probability of membership >0.90 to the shallow cluster [denoted as ‘deep (shallow origin)’]. No shallow individuals of deep-water origin were found in the U.S. Virgin Islands (denoted by nd) and no samples from Bermuda where included due to absence of patterns of depth zonation at this location. Numbers in bars indicate number of colonies assessed. USVI = U.S. Virgin Islands.

In this study, we first assessed the diversity of symbiont populations and patterns of depth zonation (if any) in *P. astreoides* by selecting a subset of corals haphazardly and using denaturing gradient gel electrophoresis (DGGE) and sequencing dominant band profiles of ITS-2 rDNA. Overall, DGGE showed evidence for strong depth zonation in algal symbionts in both Florida and the USVI, but not in Bermuda (Fig. 6a). In Florida and the USVI, most shallow and mid-depth colonies appear to only host *Symbiodinium* types A4 or A4a, whereas most deep colonies appear to only host *Symbiodinium* type C1. The shift between A4/A4a and C1 occurred at relatively deep depths, with colonies at 20–30 m hosting either A4/A4a or C1, and colonies >30 m hosting only C1 (although larger sample sizes can help clearly elucidate where this shift occurs). In Bermuda, on the other hand, all colonies hosted *Symbiodinium* type A4 or A4a across all depths (Fig. 6a). Corals from the shallow inshore site in Bermuda, however, also hosted *Symbiodinium* type B1.

Quantitative PCR assays (qPCR) were used in addition to DGGE to better understand patterns of depth zonation in Florida and the USVI, by detecting the presence of any “background” symbiont types not detectable by DGGE (e.g., Mieog *et al.*^21^), if any. Assays targeted *Symbiodinium* in clades A, C and D. Despite the low sample sizes, this method (Fig. 6b) revealed mixed symbiont communities (i.e., multiple *Symbidinium* types within a colony) in Florida shallow [(A, A (+C), A (+D), or A (+C+D)] and deep corals [(A, C or A (+C)], but not in the USVI. Furthermore, half of the shallow corals assessed from Florida had background levels of *Symbiodinium* in clade D (presumably D1a*/S. trenchi*) not previously detected with DGGE (Fig. 6b). Finally, a subset of the colonies identified as potential immigrants or as having immigrant ancestry in genetic analyses [denoted as “shallow (deep origin)” or “deep (shallow origin)” in Fig. 6b)] also hosted mixed symbiont communities in Florida, but not in the USVI (which only hosted A4/A4a or C1). Interestingly, these colonies hosted symbionts most commonly found in the habitat they settled in, rather than the symbionts most commonly found in their depth of origin (Fig. 6b).

## Discussion

The extent to which reefs are effectively connected to one another and their potential to serve as sources of larval replenishment following disturbance are topics of considerable interest in contemporary reef science. Understanding patterns of coral connectivity, sources of recruitment and recovery timelines are critical needs for managers who are increasingly operating under the implicit assumption that climate change and other impacts to reefs are unlikely to improve in the short term. In this study, we conducted the first assessment of genetic connectivity for the brooding coral *Porites astreoides* in the tropical and subtropical western Atlantic, in both horizontal and vertical directions. *P. astreoides* was expected to exhibit limited dispersal capabilities and lower genetic connectivity than broadcast spawning coral species in the region, presumably due to shorter pre-competency periods and maternal transmission of algal symbionts. However, findings revealed that *P. astreoides* (i) exhibits high levels of gene flow within the Caribbean region, (ii) can occasionally disperse and settle as far as Bermuda, (iii) exhibits patterns of vertical connectivity that vary among and within geographic locations, and (iv) displays strong patterns of depth zonation in two of the three geographic locations examined. Together, these findings suggest that *P. astreoides* is effective at dispersing both horizontally and vertically despite its brooding reproductive mode and depth zonation of algal symbionts, and exhibits a similar or greater dispersal potential compared to other Caribbean broadcast spawning taxa studied to date. Furthermore, these findings might help explain the ecological success reported for *P. astreoides* in the Caribbean compared to other scleractinian coral species^17^.

Overall, we found very little differentiation for *P. astreoides* between shallow sites in Florida and the USVI, despite being separated by >1,700 km (Figs 1, 3 and 4 and Table 1). These findings suggest that this coral species has the ability to disperse over large distances within the Caribbean/northwest Atlantic. In addition, these findings suggest high genetic exchange between the eastern and western Caribbean, compared to the lack of genetic exchange among these regions observed for broadcast spawning coral species *Acropora palmata*^22^ and *Orbicella annularis*^23^. Recent work by Holstein *et al.*^24^, however, used a modeling approach to show that *P. astreoides* consists of a highly fragmented connectivity network within the Caribbean compared to the broadcast spawning species *O. annularis*. In their model, *P. astreoides* from Florida and the USVI appear generally isolated from each other, although different findings between both studies may be explained by the different methods applied, assumptions of population connectivity, and time-scales examined. Alternatively, the lack of population subdivision between Florida and the USVI in our study could be the result of stepping-stone dispersal among locations in the fragmented connectivity network described by Holstein *et al.*^24^.

The high gene flow observed for *P. astreoides* between the USVI and Florida did not translate into high levels of connectivity between these two regions and Bermuda. Findings strongly suggest that, in contrast to *M. cavernosa*^18^, *P. astreoides* from Bermuda may be relatively isolated from the Caribbean region and northwest Atlantic (Figs 1, 3 and 4, Table 1). However, larvae originating from the Caribbean may occasionally disperse and settle in Bermuda, as suggested by the few individuals in the Bermuda shallow inshore site that were assigned to the same population common in Florida and the USVI (Fig. 1). Nunes *et al.*^25^, however, showed that, out of 6 coral species studied, *P. astreoides* was the only one with no significant differentiation between Brazil and the Caribbean, suggesting a high degree of gene flow between these two regions. Nunes and colleagues concluded that the long-distance dispersal observed in this species might be due to its ability to raft and/or its tolerance to freshwater and high sedimentation.

The degree of vertical connectivity observed for *P. astreoides* varied among and within geographic locations (Fig. 1). Within Florida, significant structure with depth was observed in all 3 regions. Patterns weakened from east to west, with the largest differentiation occurring in the Upper Keys and the lowest in the Dry Tortugas (Fig. 1). In addition, the depths at which this transition occurred were quite shallow and varied regionally: in the Upper and Lower Keys the transition occurred at ≥15 m, while in the Dry Tortugas it occurred at ≥25 m. This is not surprising, as the Dry Tortugas region has been identified as having mesoscale eddies which extend down to >100 m^26^, which may potentially act as important larval retention mechanisms^27^ and facilitate vertical movement of larvae compared to the Lower or Upper Keys. Regardless, these patterns of genetic structure with depth are in agreement with those from the Caribbean broadcast spawner species *M. cavernosa*^18^,^28^ the octocoral *Eunicea flexuosa*^29^,^30^ corals in the genus *Oculina*^31^, the brooding coral *Madracis pharensis*^32^, and the Pacific brooding coral *Seriatopora hystrix*^33^, despite different study locations, coral species and reproductive strategies.

In comparison to patterns of genetic structure observed in Florida, Bermuda and the USVI, on the other hand, appeared to be highly panmictic across depths. One possibility is that the corresponding deep habitat may be at greater depths than those assessed (>33 m) at these locations. Alternatively, deep reefs from Bermuda and the USVI may act as important local recruitment sources for their shallow water counterparts following disturbance, supporting the Deep Reef Refugia Hypothesis (reviewed in Bongaerts *et al.*^9^). In agreement with these results, biophysical modeling in the USVI found that mesophotic (>30 m) and shallow *P. astreoides* populations might be connected within one to two generations, suggesting high local connectivity^34^.

Overall, our results suggest a higher degree of interbreeding among shallow and deep *P. astreoides* colonies compared to *M. cavernosa*^18^, especially in Florida. This is not surprising, as *M. cavernosa* has to send bundles of egg and sperm to the water surface during spawning, which may reduce the potential for interbreeding if deep gametes arrive late to the surface^35^ or if there are temporal differences in spawning times^29^,^35^. Alternatively, deep and shallow *P. astreoides* colonies may have increased chances of interbreeding due to multiple reproductive events per year.

A higher number of *P. astreoides* individuals from shallow habitats in Florida also exhibited high probabilities of membership to the deep cluster compared to *M. cavernosa*, suggesting that these colonies, which originated in deep-water, were able to successfully recruit and survive in shallow habitats (Fig. 1). *P. astreoides* might be more competitive in high irradiance (shallow) habitats because it possesses symbionts in clade A (considered ‘shallow water specialists’^36^,^37^, whereas *M. cavernosa* was found to host the same *Symbiodinium* type in clade C across depths^18^, or because of availability of maternal (energy) reserves^4^ in addition to input from algal symbionts^38^. Alternatively, *P. astreoides* planulae, competent to settle a few hours after released, might be more capable of controlling their swimming and/or vertical position in the water column which might be important for selecting an optimal substratum in which to settle.

Interestingly, we found restricted gene flow between the Bermuda inshore shallow site (BDA1, see Supplementary Fig. S3) and all other Bermuda sites – including the offshore shallow site – despite similar depths and close proximity (~3.5 km). Recent work by Kenkel *et al.*^39^ also reported significant genetic differentiation among *P. astreoides* individuals from inshore vs. offshore shallow sites (2–3 m) but in the Lower Florida Keys. The authors hypothesized that *P. astreoides* coral populations inhabiting reefs <10 km apart within the same depth range can exhibit substantial physiological and genetic differences in response to thermal stress. In the present study, all of the sites in the Lower Keys can be considered offshore. However, analyses among inshore and offshore sites in the Upper Keys (see Supplementary Fig. S4) suggest that some of the genetic differences observed in Kenkel *et al.*^39^ may be attributable to whether these individuals originated in shallow vs. deep water.

Finally, although Potts and Garthwaite^40^ suggested that the two *P. astreoides* color morphs (yellow/green and brown) may represent different species, our findings show that genetic subdivision is not associated with color morph type (Fig. 5), implying that both morphs constitute a single species (i.e., are not reproductively isolated). These findings are in agreement with Weil^41^, who found no genetic differences between the two color morphs, and suggested that differences in color morph type might be driven by depth and habitat (i.e., environmental differences). Furthermore, Gleason^10^ later showed that morph-specific variation in *P. astreoides* appears to correspond to differences in UV tolerance.

To date, the role of depth zonation in algal symbionts in shaping the vertical distributions of the coral host is not well understood^16^,^30^. In this study, the characterization of algal symbionts using DGGE revealed that most *P. astreoides* shallow corals from Florida and the USVI genotyped only associated with *Symbiodinium* types A4 or A4a, while most deep colonies (particularly ≥30 m) only associated with *Symbiodinium* type C1 (Fig. 6a). Similar results were found by Baker^14^ in Panamá and Bahamas, and along depth gradients (2–25 m) in Belize^15^ and Curacao^16^. However, the depth at which we observed all colonies to host type C1 was slightly deeper than in Belize (≥30 m vs. ≤25 m)^15^. In addition, Bongaerts *et al.*^16^ reported many *Symbiodinium* profiles for *P. astreoides*, including novel ITS2 sequences in clade C not found here. These differences, however, may be a result of sampling different geographic locations and/or using different methods for the identification of algal symbiont types. Furthermore, since we were sequencing only dominant bands, it is possible that we might have found additional sub-cladal types had we sequenced all bands from each characteristic ITS2 profile.

Further analyses with quantitative PCR (Fig. 6a,b) revealed mixed algal symbiont communities in Florida’s shallow and deep colonies, as well as in colonies identified as potential immigrants or having immigrant ancestry. These colonies were more likely to host algal symbionts that matched those found in the habitats they settled in, rather than those inherited form their parents (Fig. 6b). These findings suggest that some of the *P. astreoides* colonies in Florida may have changed their symbiont communities post-settlement, either through “switching” or “shuffling” (*sensu* Baker^42^). Alternatively, colonies might have originated from a different depth and then fertilized “local” colonies, resulting in admixed individuals with the local symbionts maternally-inherited, without “switching” or “shuffling” needed. In agreement with these findings, van Oppen *et al.*^43^ observed similar patterns for the brooding coral *S. hystrix* at Scott Reef (Australia), where shallow colonies identified as having originated from deep-water also hosted the same *Symbiodinium* type most commonly found in shallow habitats.

While patterns of depth zonation were observed in Florida and the USVI, most corals in Bermuda hosted only *Symbiodinium* type A4 or A4a, regardless of depth. Corals from the inshore shallow site at this location however, also hosted *Symbiodinium* type B1 (Fig. 6a), perhaps as a result of this site’s continuous exposure to anthropogenic stressors and high sedimentation rates^44^, compared to offshore sites. Alternatively, habitat differences (e.g., inshore vs. offshore), could be driving both host (Fig. 1 and Supplementary Fig. S3) and algal symbiont differences (Fig. 6a). Regardless, the lack of depth zonation in observed in Bermuda may be the result of this site’s isolated high latitude location. Patterns of depth zonation of algal symbionts observed in Florida and the USVI, however, did not appear to affect the ability of *P. astreoides* to disperse across different depths. We hypothesize that possessing or acquiring the appropriate symbionts (‘high light’ vs. ‘low light’) might be an important mechanism used by *P. astreoides* to increase post-settlement survival across a wide range of habitats and depths. This might be particularly important for colonies inferred to be of deep-water origin that settled in shallow habitats (Fig. 6b), as only symbionts in clade A have been shown to produce UV-protective compounds^45^ that are likely to offer a competitive advantage to corals in high irradiance environments^37^.

In conclusion, we expected the Caribbean coral *P. astreoides*, with its brooding reproductive mode and maternal transmission of algal symbionts, to show low levels of gene flow in both horizontal and vertical directions. However, we found that *P. astreoides* exhibited high levels of horizontal gene flow between the USVI and Florida (>1,700 km), suggesting that *P. astreoides* has similar or greater dispersal potential compared to Caribbean broadcast spawning taxa [similar: *O. faveolata*^46^ and *M. cavernosa*^18^; greater: *A. palmata*^22^, *A. cervicornis*^47^ and *O. annularis*^46^], as well as other Caribbean brooding taxa shown to recruit in close proximity to the parent population (*Siderastraea radians*^48^; *Agaricia agaricites*^49^). Furthermore, patterns of genetic differentiation with depth observed in this study for *P. astreoides* are remarkably similar to those found for the broadcast spawning coral *M. cavernosa*^18^ in 4 of the 5 regions assessed (Upper Keys, Lower Keys, Bermuda and the USVI), despite very different life-history and reproductive strategies. In contrast, both Severance and Karl^46^ and Davies *et al.*^50^ showed significant differences in dispersal ability for congeneric species *O. annularis*/*O. faveolata*^46^ and *A. hyacinthus*/*A. digitifera*^50^ despite their similar reproductive traits. Nunes *et al.*^25^, however, found that the extent of gene flow within populations in Brazil was correlated with the reproductive traits of the species studied.

Together, results from this study have important consequences for understanding how coral reef populations might recover from stressors and how can they be managed. Our findings suggest that neither the mode of reproduction or algal transmission are necessarily good predictors of dispersal ability for coral species within the Caribbean region. Furthermore, a comparison between this and our previous study^18^ reveals that the extent of vertical gene flow is likely the result of extrinsic, site-specific factors occurring pre- or post-settlement. Since light is the primary factor limiting the maximum depth of hermatypic coral growth^51^,^52^, it is possible that the absolute depths defining the degree of genetic structure may vary as a result of site-specific variability in parameters such as water clarity and/or light intensity. Alternatively, variable oceanographic features such as those found within regions in Florida may result in restricted gene flow, indicating that populations may rely on local recruitment and thus local management is needed. Thus, future work should include a broader survey of coral species, geographic locations and larger sample sizes to better elucidate the role of shallow vs. deep coral populations as sources of larval replenishment following disturbance. In addition, newer molecular techniques (e.g., SNPs, ITS2 metabarcoding) can be used to reveal finer patterns of genetic structure for *P. astreoides* and help understand the processes affecting this species’ coral-symbiont interaction.

## Methods

### Sample collection

Field activities were focused on “shallow” (≤10 m), “intermediate” (15–20 m) and “deep” (≥25 m) coral communities along the (1) Florida Reef Tract (within sites in the Upper Keys, Lower Keys and Dry Tortugas), (2) Bermuda, and (3) the USVI (Supplementary Table S1 and Fig. S1). “Deep” reefs were defined as those ≥25 m because there is very little coral cover information available for Florida at depths ≥30 m (e.g., Murdoch and Aronson^53^; Smith *et al.*^54^), and because this depth approximates the lower 1% attenuation depth for visible radiation in the Lower Keys (~27 m^55^).

At each site, corals were sampled using SCUBA along depth transects. A haphazard approach was used to collect samples from colonies at least 1 m apart to minimize the likelihood of sampling clones. Since *P. astreoides* is known to occur as two color morphs (yellow/green and brown), we recorded this information as often as possible (N = 200). Two different sampling methods were used, per the requirements of the respective permitting agencies. Briefly, when permitted, samples were removed from colonies as small tissue biopsies (0.25 cm^2^) using a 4 mm internal diameter hollow steel punch, and preserved in 95% ethanol. Conversely, when destructive sampling was not permitted, tissue biopsies were collected using a razor blade, transferred at the surface to a 2 mL tube with 500 μL of DNAB+1% SDS^56^, and heated to 65 °C for 1.5–2 hrs. Finally, genomic DNA was extracted using the organic extraction protocol described in Rowan and Powers^56^.

### Microsatellite development

The methods used to minimize contamination of symbiont DNA in the coral used prior to 454 sequencing and library construction are described in detail in Serrano *et al.*^18^. Overall, a total 30,770 single sequence reads were generated and trimmed with PipeMeta^57^, then assembled with the GS De Novo Assembler (Roche Diagnostics Corporation, Indianapolis, IN) keeping the default settings and a minimum sequence length of 45 base pairs. Sequences were then imported to the Tandem Repeat Finder (TRF) database^58^ and processed using the default alignment parameters as follows: Match: 2; Mismatch: 7; Indels: 7. Primers were designed for a subset of sequences with a minimum of six tri-, tetra-, penta- or hexanucleotide repeats (N = 40) using the web-based program Primer 3^59^ and screened for variability by visually inspecting bands on 2% agarose gels to identify candidate markers (N = 6). Finally, specificity to host DNA was confirmed for candidate markers by screening against the algal symbionts isolated from the colony used for microsatellite development (identified as *Symbiodinium* type A4), as well other preexisting algal cultures in clades A, B, C and D isolated from this species or the coral species *O. faveolata.* None of the candidate markers amplified any of the cultured *Symbiodinium*, therefore, they were determined to be derived from the host and used in subsequent analyses.

### Microsatellite genotyping

Six microsatellite loci were further developed for scoring on an ABI 3730 automated sequencer by fluorescently labeling forward primers with NED, VIC or 6FAM (Applied Biosystems, CA). Two of the six markers exhibited strong deviations from Hardy Weinberg Equilibrium (HWE) expectations and were excluded from further analyses. PCR reactions for the remaining four loci were performed in two multiplex reactions (11 μL total volume, consisting of 2 primer pairs each) using 1 μl of 50–100 ng of template DNA and primer concentrations specific to each locus (Supplementary Table S4), 5 × PCR Reaction Buffer (Promega), 2.75 mM of MgCl2 (Promega), 0.8 mM of dNTPs, and 0.5 U of Taq polymerase (Promega). In total, *P. astreoides* samples were amplified with a total of 8 microsatellite loci: four as described above (Supplementary Table S4) and four (markers Past_3, Past16, Past_17 and Past_21) as described in Kenkel *et al.*^39^. Thermal cycling for all reactions, as well as visualization of PCR products were performed as described in Serrano *et al.*^18^. Samples that failed to amplify more than two of the eight loci (N = 190) or samples which exhibited tri-allelic genotypes (N = 12) at any of the markers from Kenkel *et al.*^39^ were excluded from further analyses. In this dataset, there was a per locus failure rate of <10% (except for marker Past_3 which had a failure rate of 13.7%), and a per sample failure rate of 0.51%.

### Analysis of multi-locus genotype (MLG) data

Identical MLGs (clones) were identified in GenAlEx v.6.41^60^ by requiring complete matches at all loci. The same number of unique MLGs (N = 590) were found whether missing data were considered or not. Tests for conformation to HWE expectations were performed using the program Genepop^61^. The R-package FDRtool was then used to adjust p-values for multiple testing^62^. Since large heterozygote deficits are common in marine invertebrates^63^,^64^, the program INEST^65^ was used to distinguish among some of the possible causes for departures for HWE by estimating null allele frequencies while accounting for inbreeding. Population level pairwise F_ST_ comparisons were performed in GenAlEx v.6.41. Finally, a Principal Component Analysis (PCA) was performed on a matrix of covariance values calculated from population allele frequencies in the program GenoDive v.2.20^66^. Then, to assess if there was a relationship between uncorrected F_ST_ and geographic distance, a Mantel’s test for isolation-by-distance was run in GenoDive with 999 bootstrap permutations, including only sampling locations with ≥10 individuals.

Population structure was investigated using a Bayesian clustering approach performed in STRUCTURE v.2.3.3^67^ on the web-based Bioportal server from the University of Oslo. Correlated allele frequencies and admixed populations were assumed. Values of K (hypothesized number of populations) from 1 to 20 were tested by running 3 replicate simulations per K with 10^6^ Markov chain Monte Carlo repetitions and 10^3^ burn-in iterations. The LOCPRIOR option was not used. A preliminary run was conducted by site to assess whether individuals within a same region and depth could be pooled to increase statistical power but this run did not introduced additional structure (see Supplementary Fig. S3). The most likely value for K based on the STRUCTURE output was then determined by plotting the log probability [L(K)] of the data over multiple runs and comparing that with delta K^68^, as implemented in the web-based program STRUCTURE HARVESTER^69^. An alternative approach, implemented in the program ObStruct^70^, was also used to statistically analyze the STRUCTURE ancestry profiles and determine whether the inferred population assignment and the factor of interest (e.g., origin of individuals) were significantly correlated (see Supplementary Table S5 and Fig. S2). The results from STRUCTURE were used as prior information to test values of K from 2 to 4 in analyses of ObStruct. Finally, results of the three STRUCTURE runs for the most likely K were then merged with CLUMPP^71^ and visualized with DISTRUCT^72^.

### Algal symbiont characterization

A subset of the corals used for microsatellite analyses was selected haphazardly to assess the diversity of symbiont populations and patterns of depth zonation in *P. astreoides*, if any. *Symbiodinium* types were identified by denaturing gradient gel electrophoresis (DGGE) and sequencing of ITS-2 rDNA using the primers ITSintfor2 and ITS2clamp^73^. Amplification products were separated by DGGE using a 35–75% gradient. Dominant bands on the gel were excised, re-amplified and sequenced using the BigDye Terminator v3.1 cycle sequencing kit and an automated sequencer (ABI 3730). Sequences were then identified via BLAST in GenBank (accession numbers are given in Supplementary Table S6).

Quantitative PCR (qPCR) assays were used in addition to DGGE in a subset of corals from Florida and the USVI to better understand patterns of depth zonation and detect the presence of “background” symbiont types not detectable by DGGE (e.g., Mieog *et al.*^21^). Assays targeted *Symbiodinium* in clades A, C and D and were validated for target specificity and amplification efficiency as described in Correa *et al.*^74^ and Cunning and Baker^75^. The assay for *Symbiodinium* clade A targeted the ITS1–5.8S-ITS2 of the large subunit of the nuclear rDNA^74^. Assays targeting specific actin loci in *Symbiodinium* clades C and D, however, were carried out in multiplex as described in Cunning and Baker^75^. All qPCR reactions were performed with StepOnePlus Real-Time PCR System (Applied Biosystems, CA) using reaction volumes of 10 μL and 1 μL of genomic DNA template. Two replicates were used per sample and clade assayed and positive amplifications were counted only when both technical replicates produced cycle threshold (C_T_) value <35 and there was no amplification in the no-template controls. Finally, potential immigrants or individuals with immigrant ancestors were previously identified in STRUCTURE as having a probability of membership >0.90 to the deep cluster [denoted as “shallow (deep origin)” in Fig. 6b], or as having a probability of membership >0.90 to the shallow cluster [denoted as “deep (shallow origin)” in Fig. 6b].

## Additional Information

**Data availability**: Porites astreoides multi-locus genotypes, sampling locations for each colony and Symbiodinium typing results from DGGE and quantitative PCR are available in Penn State’s Scholar Sphere repository at https://scholarsphere.psu.edu/collections/4t64gn20s. Structure analysis input file and settings are also available in https://scholarsphere.psu.edu/collections/4t64gn20s.

**How to cite this article**: Serrano, X. M. *et al.* Long distance dispersal and vertical gene flow in the Caribbean brooding coral *Porites astreoides*. *Sci. Rep.* **6**, 21619; doi: 10.1038/srep21619 (2016).

## Supplementary Material

Supplementary Information

## Figures and Tables

**Table 1:** *Porites astreoides* pairwise F_ST_ values for each population.

Population	UK shallow	UK mid	UK deep	LK shallow	LK mid	LK deep	DT shallow	DT mid	DT deep	BDA shallow	BDA mid	BDA deep	USVI shallow	USVI mid	USVI deep
UK shallow	0.000														
UK mid	0.041	0.000													
UK deep	**0.062**	0.012	0.000												
LK shallow	0.020	0.030	0.045	0.000											
LK mid	0.033	0.008	0.032	0.017	0.000										
LK deep	**0.058**	0.021	0.023	0.028	0.017	0.000									
DT shallow	**0.053**	**0.085**	**0.085**	**0.058**	**0.071**	**0.085**	0.000								
DT mid	0.037	**0.076**	**0.100**	0.029	0.049	**0.064**	**0.069**	0.000							
DT deep	0.029	0.014	0.021	0.024	0.005	0.018	**0.056**	0.046	0.000						
BDA shallow	**0.070**	**0.079**	**0.070**	**0.057**	**0.076**	**0.057**	**0.096**	**0.093**	**0.068**	0.000					
BDA mid	**0.070**	**0.100**	**0.091**	**0.071**	**0.100**	**0.088**	**0.106**	**0.095**	**0.081**	0.024	0.000				
BDA deep	**0.088**	**0.117**	**0.107**	**0.094**	**0.127**	**0.110**	**0.119**	**0.126**	**0.106**	0.033	0.001	0.000			
USVI shallow	0.029	0.048	**0.071**	0.033	0.033	**0.068**	**0.052**	0.045	0.031	**0.091**	**0.095**	**0.111**	0.000		
USVI mid	0.037	0.043	**0.050**	0.030	0.033	0.039	0.048	0.043	0.027	**0.077**	**0.093**	**0.118**	0.025	0.000	
USVI deep	0.032	0.038	**0.051**	0.020	0.021	0.032	**0.054**	0.048	0.026	**0.069**	**0.095**	**0.119**	0.032	0.011	0.000

Statistically significant values (p < 0.05) after FDR correction are highlighted in bold. UK = Upper Keys, LK = Lower Keys, DT = Dry Tortugas, Bermuda = Bermuda and USVI = U.S. Virgin Islands.
