# Supplementary material for: Long distance dispersal and vertical gene flow in the Caribbean brooding coral Porites astreoides
Source: Sci Rep. 2016 Feb 22;6:21619. doi: 10.1038/srep21619 (PMC4761953; doi:10.1038/srep21619)

Long distance dispersal and vertical gene flow in the Caribbean brooding coral *Porites astreoides*

Xaymara M. Serrano, Iliana B. Baums, Tyler B. Smith, Ross J. Jones, Tonya L. Shearer and Andrew C. Baker

Supplementary Information

Supplementary Table S1. *Porites astreoides* samples (N= 660). Given are total sample size (N), number of unique multi-locus genotypes (Ng) and ratio of genets over samples collected (Ng/N). GPS locations are in decimal degrees. USVI= U.S. Virgin Islands

Region	Sub region	Population	Site name or location	Site in map	Estimated depth (m)	N	Ng	Ng/N	Latitude	Longitude
**Florida**	Upper Keys	UK shallow	Conch reef	UK1	5	7	6	0.86	24.9465	-80.50207
			DL patch	UK2	5	10	10	1.00	25.0136833	-80.41387
			Little Conch reef	UK3	5	17	13	0.76	24.9511167	-80.4614
			Marker 39	UK4	5	11	11	1.00	25.0094333	-80.45792
			Sand island	UK5	5	24	19	0.79	25.0178667	-80.36823
			Tavernier Rocks	UK6	5	3	3	1.00	24.9389833	-80.56272
			Hens and Chickens	UK7	5	19	12	0.63	24.9341333	-80.54952
			Wolf reef	UK8	5	9	9	1.00	25.02185	-80.39623
			Behind Conch reef	UK9	5	18	16	0.89	24.9575833	-80.45603
		UK mid	SW of Molasses reef	UK10	16	16	14	0.88	25.0042333	-80.38757
			NE of Conch reef SPA	UK11	17	11	9	0.82	24.9465333	-80.45687
			Conch reef mid TS	UK12	20	53	42	0.79	24.94621667	-80.45595
		UK deep	Conch reef deep TS	UK13	27	18	15	0.83	24.94698333	-80.45561667
			Conch reef deep1	UK14	29	3	3	1.00	24.9580667	-80.45243
			Pickles deep	UK15	25	1	1	1.00	24.97095	-80.43075
			N of Molasses reef	UK16	37	6	5	0.83	25.0041333	-80.37987
	Lower Keys	LK shallow	Western Sambo reef	LK1	8	8	8	1.00	24.4784833	-81.7302
			Marker 32	LK2	8	18	15	0.83	24.4741667	-81.74268
			Near Key West	LK3	9	21	21	1.00	24.4687667	-81.82217
		LK mid	American shoal mid TS	LK4	14	13	10	0.77	24.5158167	-81.54248
			American shoal mid	LK5	16	22	22	1.00	24.5138167	-81.54315
		LK deep	American shoal	LK6	25	36	35	0.97	24.5042167	-81.58197
	Dry Tortugas	DT shallow	Dry Tortugas National Park	DT1	8	44	40	0.91	24.6107833	-82.87133

	DT mid	Near Dry Tortugas	DT2	15	26	26	1.00	24.72225	-82.78715
	DT deep	Outside Dry Tortugas	DT3	25	48	39	0.81	24.62875	-83.10167
**Bermuda**	BDA shallow	Castle harbour 4m inshore	BDA1	4	26	19	0.73	32.3598833	-64.69243
		Castle harbour 4m offshore	BDA2	4	22	22	1.00	32.3367167	-64.65738
	BDA mid	Castle harbour 18 m	BDA3	18	32	31	0.97	32.3354167	-64.65405
	BDA deep	Castle harbour 26 m	BDA4	26	20	18	0.90	32.3252667	-64.65423
**USVI**	USVI shallow	Flat Cay	USVI1	7	42	40	0.95	18.5303667	-65.65172
	USVI mid	Buck Island	USVI2	20	12	12	1.00	18.4647167	-65.49722
	USVI deep	College shoal	USVI3	30-33	44	44	1.00	18.3098167	-65.12772
**TOTAL**					**660**	**590**	**0.90**		

Supplementary Table S2. Summary of statistics per locus and population for *Porites astreoides*. N = number of samples genotyped, N_a = number of alleles, H_o = observed heterozygosity, H_e = expected heterozygosity, P_{hwe} = p value for tests of Hardy Weinberg Equilibrium. Seven out of 120 comparisons are significant after FDR-correction (highlighted in bold). USVI= U.S. Virgin Islands

**Region**	**Sub region**	**Depth**		**Locus**							
				**PA3**	**PA7**	**PA13**	**PA69**	**Past_3**	**Past_16**	**Past_17**	**Past_21**
**Florida**	Upper Keys	shallow	N	67	98	81	89	80	99	93	93
			Na	8	6	11	16	4	3	5	7
			Ho	0.808	0.692	0.862	0.853	0.512	0.524	0.570	0.420
			He	0.799	0.767	0.877	0.867	0.461	0.407	0.551	0.397
			Phwe	0.033	0.003	0.003	**0.000**	0.210	**0.000**	0.042	0.726
		mid	N	50	64	60	65	55	64	56	64
			Na	9	5	10	12	4	2	4	6
			Ho	0.765	0.662	0.967	0.909	0.536	0.169	0.825	0.492
			He	0.798	0.679	0.875	0.879	0.530	0.156	0.703	0.460
			Phwe	0.636	0.497	0.010	0.012	1.000	1.000	0.029	0.286
		deep	N	19	24	24	24	22	24	19	24
			Na	7	4	9	13	2	2	4	5
			Ho	0.850	0.800	0.720	0.840	0.652	0.160	0.800	0.600
			He	0.728	0.722	0.845	0.863	0.449	0.216	0.632	0.519
			Phwe	0.224	0.503	0.003	0.063	0.050	0.287	0.078	0.649
	Lower Keys	shallow	N	39	43	42	43	39	44	43	40
			Na	6	5	11	15	3	2	6	6
			Ho	0.744	0.767	0.810	0.930	0.487	0.432	0.535	0.475
			He	0.771	0.720	0.768	0.906	0.541	0.342	0.594	0.428
			Phwe	0.259	0.484	0.692	0.690	0.250	0.170	0.001	0.843
		mid	N	27	32	28	27	31	32	32	30
			Na	7	4	10	9	3	2	6	5
			Ho	0.815	0.469	0.643	0.889	0.581	0.250	0.719	0.567
			He	0.797	0.613	0.863	0.863	0.497	0.222	0.736	0.499
			Phwe	0.466	0.129	0.019	0.135	0.493	1.000	0.269	0.944
		deep	N	34	33	31	32	32	35	33	30
			Na	7	5	11	14	2	3	5	5
			Ho	0.882	0.727	0.742	0.969	0.406	0.286	0.667	0.533

			He	0.729	0.613	0.879	0.905	0.484	0.252	0.592	0.531
			Phwe	0.989	0.730	0.002	0.418	0.463	1.000	0.820	0.562
	Dry Tortugas	shallow	N	35	39	38	25	39	40	28	39
			Na	6	5	11	13	3	2	4	5
			Ho	0.730	0.683	0.825	0.963	0.610	0.595	0.767	0.561
			He	0.722	0.784	0.803	0.882	0.554	0.506	0.661	0.450
			Phwe	0.259	0.002	**0.000**	**0.000**	0.887	0.353	0.171	0.026
		mid	N	26	25	23	26	25	25	22	22
			Na	6	5	8	11	3	2	3	5
			Ho	0.815	0.808	0.500	0.963	0.538	0.462	0.304	0.522
			He	0.788	0.711	0.798	0.832	0.480	0.434	0.329	0.466
			Phwe	0.062	0.482	**0.000**	0.085	0.594	1.000	0.618	0.510
		deep	N	36	38	38	36	37	39	37	39
			Na	8	5	11	13	3	2	4	5
			Ho	0.972	0.895	0.842	0.861	0.514	0.410	0.730	0.641
			He	0.829	0.726	0.859	0.874	0.505	0.330	0.690	0.566
			Phwe	0.437	0.191	0.001	0.001	1.000	0.314	0.306	0.881
**Bermuda**		shallow	N	31	31	31	35	40	41	40	34
			Na	6	5	8	13	2	2	3	5
			Ho	0.939	0.788	0.879	0.838	0.452	0.209	0.476	0.722
			He	0.777	0.721	0.715	0.864	0.441	0.226	0.373	0.641
			Phwe	0.005	0.044	0.083	**0.000**	1.000	0.519	0.150	0.161
		mid	N	27	30	26	26	28	30	29	24
			Na	6	5	6	5	3	2	3	5
			Ho	0.892	0.725	0.833	0.889	0.632	0.450	0.051	0.647
			He	0.765	0.704	0.766	0.716	0.525	0.353	0.051	0.660
			Phwe	0.030	0.242	0.043	0.329	0.190	0.164	1.000	0.583
		deep	N	15	18	17	16	14	17	18	17
			Na	5	4	4	5	2	2	4	4
			Ho	0.824	0.700	0.789	0.722	0.500	0.368	0.150	0.737
			He	0.759	0.619	0.721	0.703	0.444	0.309	0.146	0.706
			Phwe	0.669	0.087	0.203	0.914	1.000	1.000	1.000	0.518
**USVI**		shallow	N	38	40	40	40	35	39	35	35
			Na	9	5	10	17	3	3	5	6

	Ho	0.925	0.762	0.810	0.881	0.351	0.293	0.730	0.568
	He	0.814	0.680	0.838	0.894	0.378	0.338	0.699	0.586
	Phwe	0.445	0.446	0.001	0.102	0.550	0.143	0.369	0.685
mid	N	12	12	10	12	12	12	11	12
	Na	7	4	6	8	3	2	5	3
	Ho	0.750	0.833	0.900	0.750	0.333	0.667	0.636	0.500
	He	0.804	0.634	0.842	0.841	0.598	0.464	0.615	0.453
	Phwe	0.391	0.478	0.252	0.101	0.100	0.216	0.241	0.341
deep	N	40	38	39	35	41	43	39	41
	Na	9	5	9	14	3	3	6	5
	Ho	0.875	0.526	0.795	0.829	0.561	0.395	0.590	0.341
	He	0.762	0.552	0.854	0.925	0.539	0.353	0.654	0.304
	Phwe	0.140	0.159	0.002	**0.000**	1.000	0.729	0.221	1.000

Supplementary Table S3. Mean null alleles and inbreeding coefficient per locus and population for *Porites astreoides*. USVI= U.S. Virgin Islands

**Region**	**Sub region**	**Depth**	**Mean null allele frequency**								**Fi**		
			**PA3**	**PA7**	**PA13**	**PA69**	**Past_3**	**Past_16**	**Past_17**	**Past_21**	**Mean**	**Lower CI**	**Upper CI**
**Florida**	Upper Keys	shallow	0.03	0.02	0.02	0.02	0.02	0.04	0.02	0.03	0.00	0.00	0.02
		mid	0.04	0.05	0.02	0.03	0.03	0.04	0.01	0.01	0.00	0.00	0.02
		deep	0.04	0.14	0.04	0.04	0.04	0.04	0.08	0.04	0.01	0.00	0.04
	Lower Keys	shallow	0.07	0.04	0.07	0.04	0.05	0.04	0.03	0.02	0.01	0.00	0.05
		mid	0.05	0.07	0.04	0.04	0.04	0.10	0.12	0.04	0.01	0.00	0.06
		deep	0.09	0.06	0.03	0.06	0.02	0.03	0.09	0.02	0.01	0.00	0.04
	Dry Tortugas	shallow	0.03	0.04	0.03	0.03	0.04	0.08	0.05	0.03	0.01	0.00	0.03
		mid	0.06	0.07	0.10	0.05	0.06	0.04	0.18	0.02	0.01	0.00	0.04
		deep	0.06	0.04	0.04	0.04	0.02	0.02	0.04	0.03	0.01	0.00	0.03
**Bermuda**		shallow	0.05	0.08	0.04	0.03	0.02	0.05	0.02	0.03	0.01	0.00	0.04
		mid	0.00	0.00	0.00	0.00	0.00	0.01	0.00	0.00	0.00	0.00	0.03
		deep	0.09	0.08	0.11	0.06	0.05	0.05	0.05	0.07	0.01	0.00	0.06
**USVI**		shallow	0.02	0.03	0.00	0.00	0.00	0.00	0.00	0.00	0.01	0.00	0.03
		mid	0.19	0.07	0.09	0.10	0.09	0.06	0.07	0.08	0.01	0.00	0.08
		deep	0.05	0.05	0.07	0.05	0.02	0.06	0.06	0.06	0.01	0.00	0.05

Supplementary Table S4. Newly-developed microsatellite loci for *Porites astreoides*. Given are the locus name, primer sequences, repeat type followed by the number of repeats and the size range of the alleles amplified in base pairs (bp). All reactions had the same annealing temperature (57°C). Forward primers were fluorescently labeled with one of three dyes (6FAM, VIC or NED). Loci were amplified in two multiplex reactions (plex A and B) or as described in Kenkel et al.[31].

Locus	Primer sequence (5'-3')	Motif type	Allele size range (bp)	Forward primer (µM)	Reverse primer (µM)	Plex
**PA3**	F: VIC-CATTAACCGACTACAGTCCGT<br>R: ACGTAAATCGCAGGACCTC	(TTCTT) imperfect	328-368	0.08	0.08	A
**PA7**	F: 6FAM-TTACAGTGGTCAAGCCTGG<br>R: TTACAGGCTCCCACACTAGC	(CGTC)2 CATC (CGTC)6	244-268	0.4	0.4	B
**PA13**	F: NED-AGATCCGCCAAGGCGAGTT<br>R: GAGCGACGTAGGCGCAAAGAT	(ATT)2 ATG (ATT)9	162-219	0.4	0.4	B
**PA69**	F: 6FAM-GCCTACCATGTTAAATCCTTG<br>R: TGGTGTAAGTGAAGGTCACA	(ATT) imperfect	157-196	0.08	0.08	A

Supplementary Table S5. R^2 values calculated using ObStruct for STRUCTURE analysis of *Porites astreoides* samples. ObStruct was run assuming two, three and four populations (K = 2 – 4). A high R^2 value indicates strong diversification and/or population structure (Gayevskiy et al. 2014). In the present dataset, the R^2 value is highest when data is partitioned by geographic location (Bermuda vs. Florida and the USVI), and by depth (within Florida), suggesting that the "optimal" number of populations is three (K = 3). For visualizations of the structure, please refer to Supplementary Figure S2 (K = 3). *Denotes a p-value <0.0001

Number of populations inferred (K)	R^2
**2**	0.50*
**3**	0.59*
**4**	0.50*

Supplementary Table S6. *Symbiodnium* types identified in figure 5 and corresponding GenBank accession numbers for the ITS-2 marker.

***Symbiodinium* taxa**	**GenBank accession numbers**
**A4/A4a**	EU449033/EU449040
**C1**	JQ180021
**D1a**	AF499802
**B1**	FJ811928

Supplementary Figure S1. Sampling locations in the Caribbean and western Atlantic. Individual sites are labeled as designated in Table S1. White circles denote shallow (≤10 m) sites, gray circles denote intermediate (15 – 20 m) sites, and black circles denote deep (≥ 25 m) sites. Maps were created using ArcGIS version 10.2 by Esri.

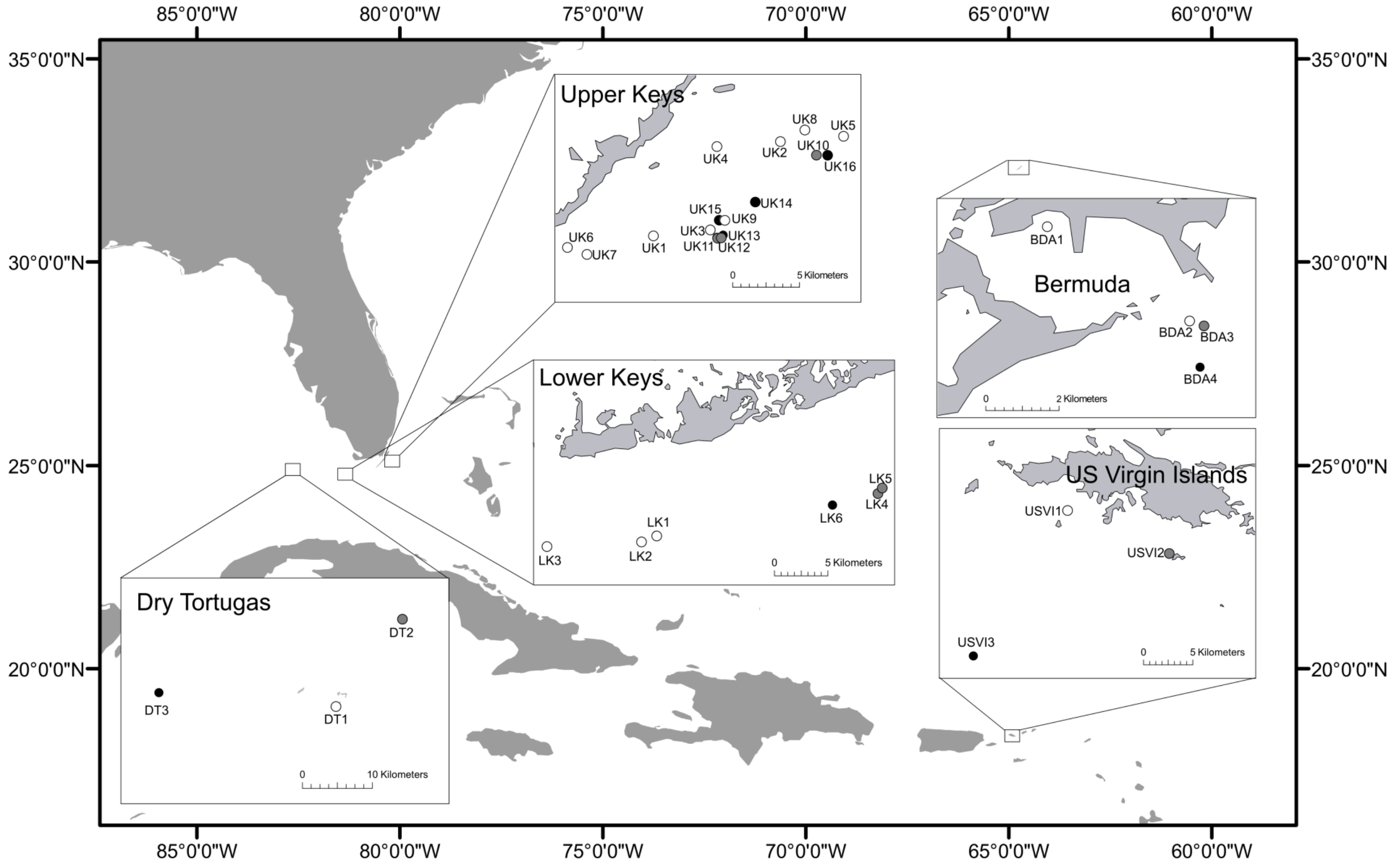

Supplementary Figure S2. Canonical discriminant analysis (CDA) calculated using ObStruct for *Porites astreoides* samples. ObStruct was run assuming three populations (K = 3). (a) Median and 50% ellipse for each predefined population (see below). The inner grey ellipse contains 50% of all individuals while the outer grey ellipse contains 95% of all individuals. This visualization provides an indication of the separation of populations and the within group variation. (b) Hypothesis-error (HE) plot showing the variation in the group means on two variables relative to the error variance. The colored arrows indicate the position of the inferred populations relative to the axes obtained by the CDA. For (a) and (b), the numbers in black indicate predefined populations (1 = UK shallow, 2 = UK mid, 3 = UK deep, 4 = LK shallow, 5 = LK mid, 6 = LK deep, 7 = DT shallow, 8 = DT mid, 9 = DT deep, 10 = BDA shallow, 11 = BDA mid, 12 = BDA deep, 13 = USVI shallow, 14 = USVI mid, 15 = USVI deep) and for (B), the numbers in blue next to the arrows indicate inferred populations. Overall, this method suggests that the "optimal" number of populations present in this dataset is three (K = 3, see Table S4).

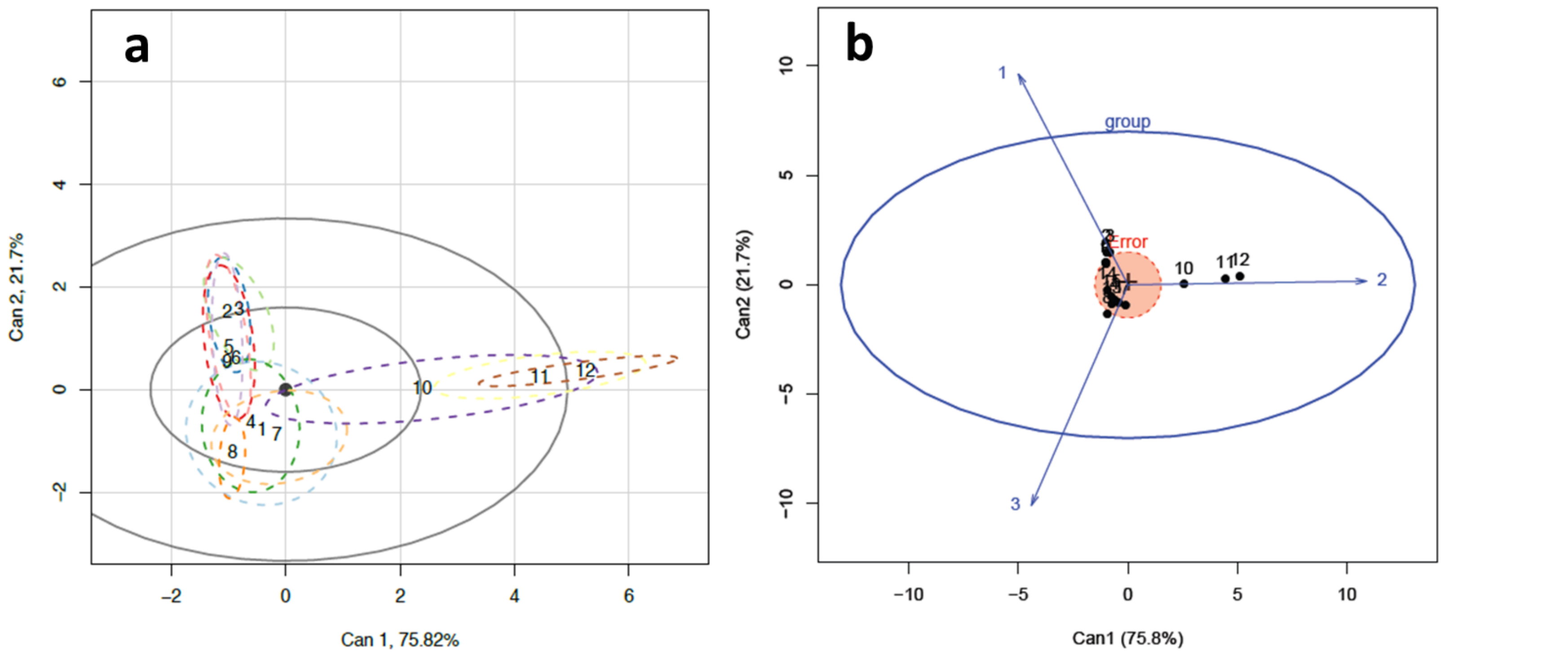

Supplementary Figure S3. *Porites astreoides* population structure by site (as designated in Table S1 and Figure S1). Correlated allele frequencies and admixed populations were assumed. Values of K from 1 – 20 were tested by running 3 replicate simulations per K (hypothesized number of populations) with 10^6 Markov Chain-Monte Carlo repetitions and 10^3 burn-in iterations. Bar graphs show the average probability of membership (y-axis) of individuals (N = 590, x-axis) in K = 3 clusters as identified by STRUCTURE.

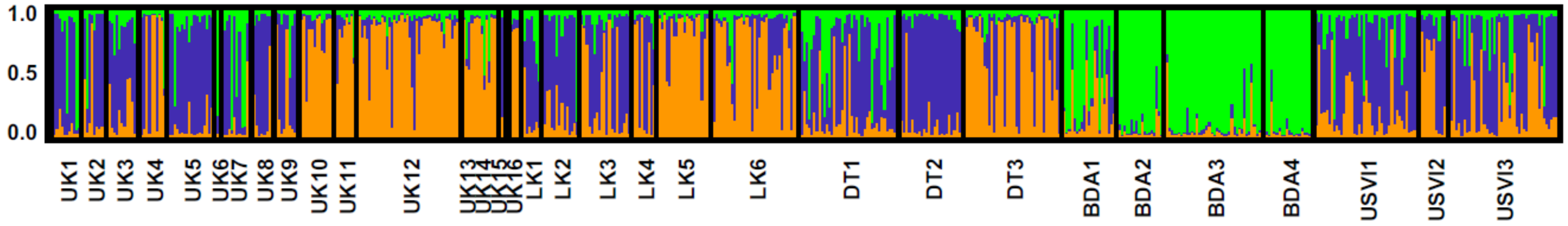

Supplementary Figure S4. *Porites astreoides* population structure of individuals from the Upper Keys (Florida), designated as either inshore [shallow (≤10 m)] or offshore [shallow (≤10 m), mid (15-20 m) or deep (≥25 m)]. Sites UK6 and UK7 were considered inshore sites, whereas sites UK3, UK5, UK9 and UK10-16 were considered offshore sites (site names and GPS locations are given in Table S1). Bar graphs show the average probability of membership (y-axis) of individuals (N = 152, x-axis) in K = 2 clusters as identified by STRUCTURE. Samples were arranged in order of increasing depth.

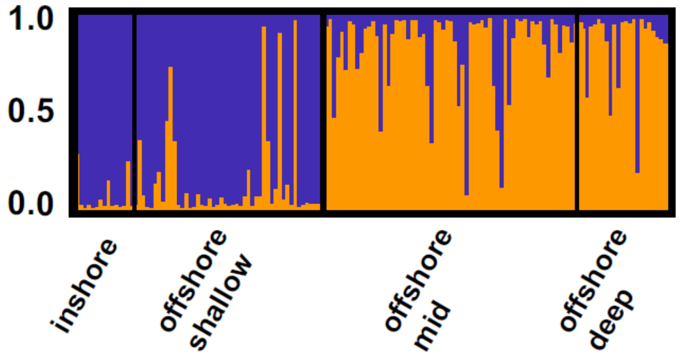

Supplement: Supplementary Information [file srep21619-s1.pdf]
